# Experimental Nanovaccine Offers Protection Against Repeat Exposures to *Trypanosoma cruzi* Through Activation of Polyfunctional T Cell Response

**DOI:** 10.3389/fimmu.2020.595039

**Published:** 2020-12-22

**Authors:** Imran H. Chowdhury, Nandadeva Lokugamage, Nisha Jain Garg

**Affiliations:** ^1^ Department of Microbiology and Immunology, The University of Texas Medical Branch (UTMB), Galveston, TX, United States; ^2^ Institute for Human Infections and Immunity, UTMB, Galveston, TX, United States

**Keywords:** *Trypanosoma cruzi*, Chagas disease, nanovaccine, T cell response, repeat infection

## Abstract

A parasitic protozoan *Trypanosoma cruzi* (*T. cruzi*) is the etiologic agent of Chagas disease. Previously, we have identified *T. cruzi* antigens TcG2 and TcG4 as potential vaccine candidates, cloned in eukaryotic expression vector pCDNA3.1 (referred as p2/4) and tested their ability to elicit protection from *T. cruzi* infection. In the present study, we subcloned the two antigens in a nanoplasmid that is optimized for delivery, antigen expression, and regulatory compliance standards, and evaluated the nanovaccine (referred as nano2/4) for prophylactic protection against repeat *T. cruzi* infections. For this, C57BL/6 mice were immunized with two doses of p2/4 or nano2/4 at 21 days interval, challenged with *T. cruzi* 21 days after 2^nd^ immunization, and euthanized at 10- and 21-days post-infection (pi) corresponding to parasite dissemination and replication phase, respectively. Some mice were re-challenged 21 days pi and monitored at 7 days after re-infection. Without the help of a vaccine, *T. cruzi* elicited delayed and sub-par T cell activation and low levels of effector molecules that failed to control tissue dissemination and replication of the parasite and provided no protection against repeat challenge infection. The nano2/4 was most effective in eliciting an early activation and production of IFN-γ by CD4^+^T effector/effector memory (T_EM_) cells and cytolytic perforin (PFN) and granzyme B (GZB) molecules by CD4^+^ and CD8^+^ T_EM_ subsets at 10 days pi that was followed by robust expansion of CD4^+^ and CD8^+^ T_EM_ and T_CM_ cells with further increase in IFN-γ production at 21 days pi. Consequently, nano2/4-immunized mice exhibited potent control of parasite dissemination at 10 days pi, and tissue parasite burden and tissue inflammatory infiltrate and necrosis were barely detectable at 21 days pi. Furthermore, nano2/4-immunized mice responded to re-challenge infection with high levels of effector molecules production by CD4^+^ and CD8^+^ T_EM_ subpopulations that offered even better control of tissue parasite burden than was observed after 1^st^ infection. In comparison, non-vaccinated/infected mice exhibited clinical features of sickness and 59% mortality within 7 days after re-infection. In conclusion, we show that delivery of TcG2 and TcG4 in nanoplasmid offers excellent, protective T cell immunity against repeat *T. cruzi* infections.

## Introduction

*Trypanosoma cruzi* (*T. cruzi* or *Tc*) is the etiological agent for Chagas disease (CD). The prolonged burden of CD primarily occurs in Latin American countries, Mexico, and USA (1), though CD is now recognized as a major health issue in other countries including Canada, Japan, Europe, and Australia due to migration of infected individuals from endemic to non-endemic countries (2). Individuals living in endemic countries are repeatedly exposed to the parasite, though it is not clear if immune system triggered by repeat exposures provides protection from severity of infection and disease (3).

Studies in susceptible and resistant experimental models of CD and in Chagas patients exhibiting variable severity of heart disease have indicated that sub-par innate immune responses, mixed type 1/type 2 cytokines and delayed kinetics of T lymphocytes activation and cytotoxicity allow parasite dissemination, intracellular replication, and persistence [reviewed in (4, 5)]. An important implication of these findings is that if a vaccine can be designed to elicit Th1 cytokines, trypanolytic antibodies, and concerted activities of phagocytic cells (e.g., macrophages), CD4^+^ T helper cells, and cytotoxic CD8^+^ T lymphocytes, then it would be successful in controlling parasite dissemination and replication and thereby prevent the consequent tissue damage and clinical severity of CD during the chronic phase of disease development [discussed in (6–8)].

Potential subunit vaccines are suggested to incorporate antigens expressed in trypomastigote form and amastigote form of the parasite (6, 7). This is because a vaccine designed to attack infective trypomastigote form is envisioned to prevent or limit the invasion and dissemination of the parasite to tissues. Likewise, a vaccine offering protection against intracellular replicative amastigote form of the parasite is expected to inhibit parasite replication in the cells and release in the bloodstream and thereby prevent reinfection of healthy cells and tissues. We also propose that antigens included in a vaccine should be expressed in all lineages of the parasite and such a vaccine should provide prophylactic as well as therapeutic efficacy against multiple parasite isolates circulating in the endemic countries (6, 7).

During the last decade, our team has employed bioinformatics and biological approaches to screen the *T. cruzi* genome for the identification of the potential vaccine candidates. Our studies have led to the selection of TcG2 and TcG4 antigens for further development as a candidate vaccine against *T. cruzi*. These antigens were selected because TcG2 and TcG4 were found to be expressed in infective and intracellular stages of multiple, clinically important *T*. *cruzi* strains (9, 10) and recognized by anti-parasite antibodies and T lymphocytes in infected host (9, 10) and, most importantly, when delivered as a subunit DNA vaccine, these antigens provided significant protection from chronic parasite persistence associated cardiac pathology in mice and dogs (11–13). Several other antigenic candidates have also been tested for their prophylactic and therapeutic efficacy against Chagas disease by other investigators, and the findings from these vaccine development efforts are encouraging and summarized in recent reviews (6, 7).

Nano eukaryotic expression plasmids have been developed as next generation vectors for effective, safe, and economical delivery of the genes. The nanoplasmids are small vectors that tend to utilize synthetic eukaryotic mRNA leader and terminators to limit the homology of the vector DNA sequence with the human genome and reduce the potential risk of its integration into host chromosome by homologous recombination. Some of the nanoplasmids utilized RNA-OUT (instead of antibiotic) to offer antibiotic-free approach for selection and amplification of the recombinant DNA (14), and opted for a R6K-derived mini-origin (300 bp) and an optimized SV40-CMV-HTLV-1 R chimeric promoter-intron to drive high levels and stable expression of target genes in the mammalian host cells (15). Overall, nanoplasmids offer optimized delivery vehicle backbone for increased expression of the cloned antigens at low cost and are designed in agreement with the regulatory compliance standards.

In this study, we have cloned the genes encoding *T. cruzi* TcG2 and TcG4 antigens into a NTC9385R-MCS nanoplasmid and used the recombinant plasmids as a nano2/4 vaccine. As re-infection might provoke tissue inflammation due to insults from higher parasite burden and intensify tissue necrosis in the host (16, 17), we aimed to determine if the nano2/4 vaccine provides protection against re-exposures of *T. cruzi*. For this, we immunized the C57BL/6 mice with two doses of nano2/4 and challenged with *T. cruzi* at day 21 and day 42 post-vaccination. Mice immunized with pcDNA3.1-*TcG2* and pcDNA3.1-*TcG4* (referred as p2/4) were included as controls so that we can study the effects (positive or negative) of the changes in the vector backbone on the immunogenic potential of the selected candidates against repeated *T. cruzi* infections. We examined whether and how nano2/4 modulated the systemic CD4^+^ and CD8^+^ T cell immunity against *T. cruzi* repeat infections, and whether the nano2/4 induced immunity was effective in arresting systemic parasitemia, prevent tissue invasion and replication, and parasite-induced tissue inflammation and injury.

## Materials and Methods

### Ethics Statement

We followed the National Institutes of Health guidelines for housing and care of laboratory animals. All animal experiments were conducted in accordance with the protocols that were approved by the Institutional Animal Care and Use Committee (protocol number 08-05-029) at the University of Texas Medical Branch at Galveston. All experiments were conducted in ABSL2/BSL2-approved laboratory and all personnel have received appropriate ABSL2/BSL2 training.

### Vaccine Constructs

The sequences for cDNAs for *TcG2* and *TcG4* are deposited in the GenBank (AY727915 and AY727917, respectively). The full-length cDNAs for the two antigens were previously cloned into pCDNA3.1 eukaryotic expression plasmid and the recombinant plasmids were purified by anion exchange chromatography by using a Qiagen Endo-free maxi prep kit (Qiagen, Chatsworth, CA) (9, 10). A CMV promoter-based NTC9385R-MCS nanoplasmid (Nature technology, Lincoln, NE) was utilized for sub-cloning the full-length genes encoding for TcG2 and TcG4 (18). The recombinant nanoplasmids NTC9385R-*TcG2* and NTC9385R-*TcG4* were sequenced to confirm the orientation and open reading frame, and purified by using the HyperGRO fermentation process (18).

### Mice, Immunization, and Challenge Infection With *T. cruzi*

Mouse myoblast C2C12 cells (ATCC CRL-1772) were propagated in RPMI 1640 medium containing 10% fetal bovine serum (FBS), 100 U/ml penicillin, 100 μg/ml streptomycin, and 0.1 mM sodium pyruvate. Trypomastigotes of *T. cruzi* SylvioX10/4 strain were amplified in vitro in C2C12 cells (19).

C57BL/6 mice (6-week-old) were purchased from Jackson laboratory (Bar Harbor, ME), and randomly distributed in four groups: (1) mock treatment/mock infection; (2) mock treatment/*T. cruzi* infection; (3) two doses of p2/4 followed by *T. cruzi* infection; and (4) two doses of nano2/4 followed by *T. cruzi* infection. The size of the backbone of NTC9385R nanoplasmid is approximately half of the size of backbone of eukaryotic expression plasmid pcDNA3.1. To compensate for the differences in backbone size and deliver similar amounts of antigen-encoding DNA, the vaccine referred as p2/4 was constituted of 25 µg each of pCDNA3.1-*TcG2* and pCDNA3.1*-TcG4* and the vaccine referred as nano2/4 was constituted of 12 µg each of NTC9385R-*TcG2* and NTC9385R-*TcG4*. Both vaccines were constituted in 50 µl PBS and delivered by intramuscular injection in the hind thighs in two doses at 21 days interval. Mice in groups 2, 3, and 4 were challenged with *T. cruzi* trypomastigotes (10,000 parasites per mouse) via intraperitoneal route at 21 days after the 2^nd^ immunization and euthanized on day 10 and day 21 post-infection (pi). The rational for choosing 10 days and 21 days pi was to check the efficacy of the vaccine against parasite dissemination and replication, respectively, based on our previous published studies (20).

**Figure 1:**
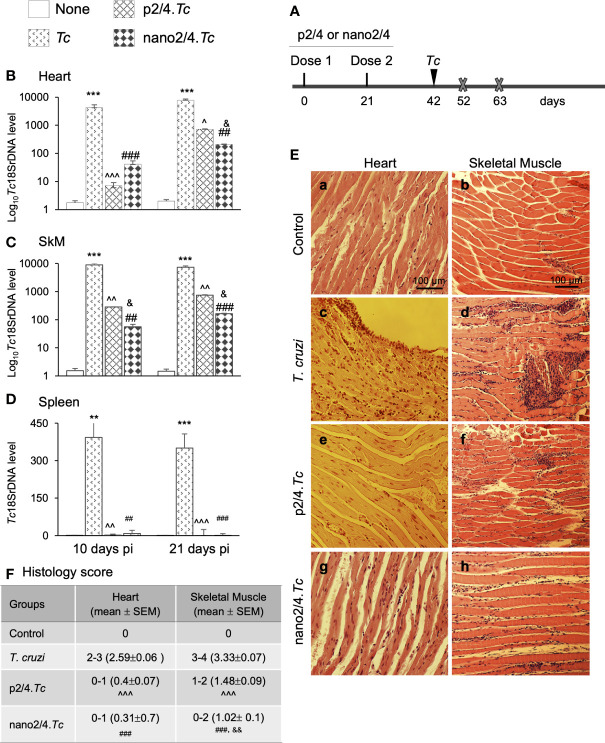
Prophylactic efficacy of nanovaccine against acute parasitemic phase. **(A) **Schematic of experimental design. C57BL/6 mice were immunized with p2/4 or nano2/4 experimental vaccines on day 0 and day 21, and infected with *T. cruzi* (SylvioX10 trypomastigotes, 10,000 parasites per mouse) on day 42. Non-infected mice were used as controls. Mice were euthanized (marked by a cross) on day 52 and day 63 corresponding to day 10 and day 21 post-infection (pi), respectively. **(B**–**D) **Tissue parasite burden. Total DNA was isolated from heart **(B)**, skeletal muscle **(C)**, and spleen **(D)** tissue at day 10 and day 21 pi, and real-time qPCR was performed to measure *Tc18SrDNA* levels (normalized to murine *Gapdh*). Data (mean ± SEM) are representative of duplicate observations per sample (*n* ≥ 5 mice per group per experiment). **(E, F) **Tissue inflammatory infiltrate in acutely infected mice (± nano vaccine). Paraffin-embedded 5-µm tissue sections were obtained at 21 days pi and examined by hematoxylin/eosin staining. Shown in **(E)** are representative images of the heart and skeletal muscle tissue sections from non-vaccinated/non-infected (a, b)****, non-vaccinated/*T. cruzi-*infected (c, d)****, p2/4.*Tc* (e, f)****, and nano2/4.*Tc* (g, h)**** groups of mice (magnification: 20×). Inflammatory score was calculated as described in Materials and Methods, and presented in **(F)**. Significance was calculated by student’s t-test (* controls vs. *Tc* only) and one-way ANOVA/Tukey’s (^^^ *T. cruzi* only vs. p2/4.*Tc*, ^#^ *T. cruzi* only vs. nano2/4*.Tc* and ^&^ **p2/4.*Tc* vs. nano2/4.*Tc*). One, two, and three symbol characters were used to annotate the p values of <0.05, <0.01, and <0.001, respectively.

To examine the vaccine efficacy against re-exposure, some mice in groups 2, 3, and 4 were re-challenged on day 21 after first infection and euthanized 7 days after the second parasite exposure. Mice were daily monitored for mortality during the study period. Sera and splenocytes were obtained for evaluating the cytokines and T cell responses in vaccinated and non-vaccinated mice after infection and reinfection. Heart, skeletal muscle, and spleen tissues were collected to monitor the parasite burden and histopathology. The schematic timeline of immunization, infection and re-challenge is presented in **Figures 1A** and **4A**.

### Parasite Burden

To evaluate the parasite burden at various stages of infection and reinfection, total DNA was purified from spleen, heart and skeletal muscles tissues by standard procedures. Total DNA (50 ng) was used as template and added to the reaction mix consisting SYBR Green Supermix (Bio-Rad) and oligonucleotides specific for *Tc*18SrDNA (forward, 5′-TTTTGGGCAACAGCAGGTCT-3′; reverse, 5′-CTGCGCCTACGAGACATTCC-3′; amplicon size: 199 bp) or murine *Gapdh* (forward, 5′-AACTTTGGCATTGTGGAAGG-3′; reverse, 5′-ACACATTGGGGGTAGGAACA-3′; amplicon size: 223 bp), and a real-time quantitative PCR was performed for 40 cycles on an iCycler thermal cycler. The threshold cycle (*C_T_*) values for *Tc18SrDNA* were calculated and normalized to *Gapdh* reference cDNA. The relative burden of *T. cruzi* in each sample was calculated by the following formula: $2^{-\Delta Ct}[2^{\left(-\Delta Ct\;sample\right)}/2^{\left(-\Delta Ct\;of\;control\right)}]$ as previously described by us (21).

### Histology

Heart and skeletal muscle tissue sections were fixed by incubation in 10% buffered formalin for 24 h and dehydrated in absolute alcohol. Tissue samples were then cleared in xylene and embedded in paraffin. Slides containing 5-micron paraffin-embedded tissue sections were stained with hematoxylin (stains nuclei blue) and eosin (stains extracellular matrix and cytoplasm pink). The H&E stained tissue slides were imaged at 20 × magnification by using an Olympus BX-15 microscope (Center Valley, PA) equipped with digital camera and Simple PCI software (v.6.0, Compix, Sewickley, PA). The inflammatory infiltrate and tissue damage were scored as described previously (22). Briefly, scoring was defined as (0) - absent/none, (1) - focal or mild with ≤1 foci, (2) - moderate with ≥2 inflammatory foci, (3) - extensive with generalized coalescing of inflammatory foci or disseminated inflammation, (4) - severe with diffused inflammation, interstitial edema, and loss of tissue integrity (22). For each group, data were captured from at least three mice, two slides per tissue, and ten microscopic fields per slide, and presented as mean value ± standard error mean (SEM).

### Flow Cytometry Analysis of Splenic T Cell Response

Spleen samples were macerated, and single-cell suspensions of spleen cells were washed with flow cytometry staining buffer (00-4222-26, eBioscience, San Diego, CA) and incubated for 10 min with Fc Block (anti-CD16/CD32; BD Pharmingen). Splenocytes (5 × 10^4^/50 ml) were incubated in dark with the fluorochrome-conjugated monoclonal antibodies to surface molecules (concentration determined by titration) for 30 min at 4°C, washed twice in cold staining buffer, fixed and permeabilized by incubation with fixation and permeabilization solution (BD Biosciences, San Jose, CA) for 20 min, and washed with perm wash buffer (BD Biosciences) (23). For analyzing intracellular molecules, cells were stained with monoclonal antibodies against cytokine (IFN-γ) or cytotoxic molecules perforin (PFN) and granzyme B (GZB) for 30 min, washed, and resuspended in staining buffer. All samples were visualized and analyzed using the 10-color BD LSRII Fortessa flow cytometer. As controls, tubes containing unstained cells and cells incubated with isotype matched IgG, live/dead stain and FMO controls were included. Data were acquired and analyzed by using a FlowJo software (v.10.5.3; TreeStar, San Carlo, CA) (18). All antibodies are listed in **Table S1**.

To establish parameters for distinguishing T cell subpopulations, we generated concatenated fcs file from all samples of the four groups. The concatenated file was filtered to remove dead/doublet cells and select on an average 1 × 10^5^ of CD3^+^ T live cells per sample by FlowJo Downsample platform. The resultant cumulative dataset was analyzed by FlowSOM FlowJo plugin, with the grid dimensions set to 7 × 7 that created a minimum spanning tree with 49 nodes (**Figure S1A**) distributed to 10 metaclusters named P0–P9 (**Figure S1B**) (24, 25). Heatmap (**Figure S1C**) was constructed to capture the identity of the T cell metaclusters based on the expression levels of the five surface markers, presented as relative median fluorescence intensity (MFI) in **Figure S1D**. We also utilized cumulative dataset to generate common t-distributed stochastic neighbor embedding (t-SNE) (**Figure S1E**) using parameters: iteration = 1,000, perplexity = 30, and learning rate (eta) = 4900 that allows reduction of dimensionality and is well suited for the visualization of high-dimensional datasets (26, 27).

To identify the changes in T cell subsets in treatment-specific manner, fcs files were individually filtered to remove dead/doublet cells and select 1 × 10^5^ of CD3^+^ T cells. Parameters defined in cumulative FlowSOM metacluster map (**Figure S1B**) were then applied to individual files to identify treatment- and time-specific changes in T cell subsets. Likewise, parameters defined in cumulative t-SNE map (**Figure S1E**) were applied to generate treatment- and time-specific t-SNE maps.

T effector/effector memory (T_EM_) and T central memory (T_CM_) sub-populations were further examined for median fluorescent intensity for intracellular IFN-g cytokine and PFN and GZB cytotoxic activity markers.

### Cytokines Release

Serum levels of cytokines, including TNF-α, IFN-γ, IL-1β, and IL-6, were measured by using sandwich ELISA kits according to the instructions provided by the manufacturer (Invitrogen).

### Statistical Analysis

All datasets were acquired from a minimum of five mice per group (minimum of duplicate observations per mouse per experiment) and managed in GraphPad Prism5 software. Data are presented as mean value ± SEM. Significance was analyzed by Student’s *t* test (comparison of 2 groups) and one-way analysis of variance (ANOVA) with Tukey’s post-hoc test or Kruskal–Wallis H/Dunn’s post-hoc test (comparison of multiple groups). Unless specified otherwise, significance is annotated in figures as ^*^ *T. cruzi* vs. control, **^^^** *T. cruzi* vs. p2/4.*Tc*, **^#^** *T. cruzi* vs. nano2/4.*Tc*, and ^&^p2/4.*Tc* vs. nano2/4.*Tc*, and one, two, and three symbol characters were used to represent the *p*-values of ≤0.05, ≤0.01, and ≤0.001, respectively.

## Results

### Prophylactic Efficacy of Nanovaccine Against Acute Dissemination and Replication of *T. cruzi*

Schematics of immunization and challenge protocol is presented in **Figure 1A**. Briefly, mice were immunized at 21 days interval with two doses of p2/4 or nano2/4 encoding TcG2 and TcG4. Mice were challenged on day 42 and euthanized on day 10 and day 21 post-infection corresponding to the early and acute parasitemic phase, respectively.

We first determined the nanovaccine efficacy by qPCR evaluation of tissue parasite burden. Mice infected with *T. cruzi* exhibited very high levels of parasite burden in heart and skeletal tissue and lower levels of parasite burden in spleen at day 10 and day 21 post-infection (**Figures 1B–D**). In comparison, mice given prophylactic p2/4 or nano2/4 exhibited a significant decline in heart, skeletal, and splenic tissue levels of parasite burden at 10 days (101–597 fold, 31–159 fold, and 46–103 fold decline, respectively) as well as at 21 days (10–37 fold, 10–44 fold, and 50–120 fold, respectively) post-infection (**Figures 1B–D**, all ^^,#^ *p* <0.01). The nano2/4 exhibited better efficacy in controlling tissue dissemination and replication of the parasite than was noted with p2/4 vaccine (**Figures 1B–D**, ^&^p < 0.05).

The histological examination of the tissue sections revealed extensive inflammatory infiltrate in the heart (histology score: 2.59) and skeletal muscle (histology score: 3.33) of infected (vs. non-infected) mice at 21 days pi (**Figures 1E.a–d, 1F**). While myocardial inflammation was more diffused, skeletal tissues exhibited diffused as well as localized large foci of inflammatory infiltrate in infected mice (**Figures 1E.c, d**). Importantly, p2/4 and nano2/4 immunization led to notable control of infiltrating cells in the heart tissue (histology score: 0.31-0.4, ^^,#^ *p* < 0.001) and skeletal muscle (histology score: 1.02–1.48, ^^,#^ *p* < 0.001) (**Figures 1E, compare c and d with e–h, 1F**). Slightly better efficacy of nano2/4 (compared to p2/4) was observed in reducing the skeletal tissue inflammatory infiltrate (^&^p < 0.01) and preserving the tissue integrity in infected mice. The uninfected control mice exhibited no detectable parasite DNA and inflammatory infiltrate in their tissues (**Figures 1B–F**). These results suggest that the TcG2/TcG4 based subunit vaccine (nano2/4>p2/4) offered protection from *T. cruzi* infection and acute parasitemic phase and consequently reduced the tissue inflammatory infiltrate in infected mice.

### T Cell Response at Early and Peak Parasitemic Phase of *T. cruzi* Infection (± Nanovaccine)

We enumerated the T cell profile in mice at 10 days pi to examine if vaccine triggers the T cell activity immediately after infection and at 21 days pi to examine if nanovaccine enhances the potency of T cell activity against replicating parasite. For this, splenocytes of all groups of mice were analyzed by flow cytometry as detailed in Materials and Methods.

**Figure 2:**
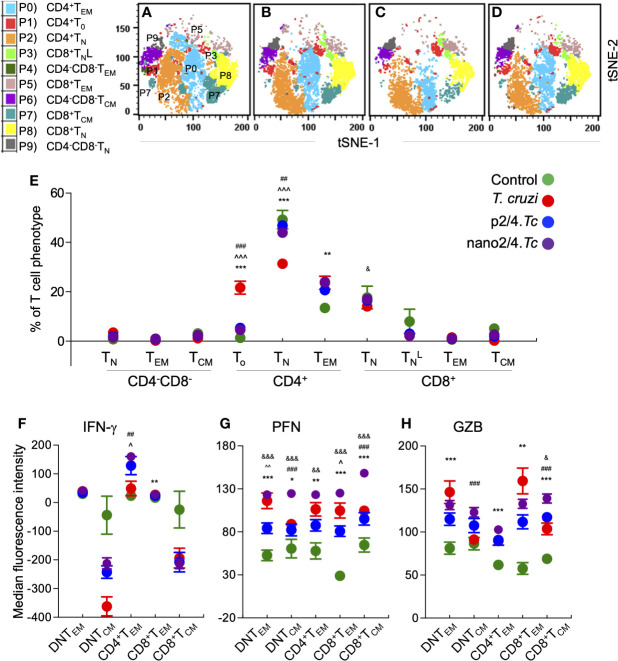
Flow cytometry analysis of T cell profile in mice at early phase of *T. cruzi* infection (± nanovaccine). C57BL/6 mice were immunized, infected, and euthanized on day 10 pi, as described in **Figure 1A**. Splenocytes were labeled with fluorescent-conjugated antibodies and analyzed by flow cytometry. FlowSOM analysis of CD3^+^ splenic T cells of mice based on the expression levels of CD4, CD8, CD25, CD62L, and CD44 antigens generated self-organizing ten meta-clusters (referred as P0–P9). **(A–D)** The tSNE (t-distributed stochastic neighbor embedding) plots were generated with non-linear reduction method to visualize all the 10 sub-populations in two-dimensional space in non-vaccinated/non-infected **(A)**, non-vaccinated/infected **(B)**, p2/4.*Tc* **(C)**, and nano2/4.*Tc* **(D)** groups of mice. **(E)** Bar graph shows P0–P9 sub-populations of T cells in all four groups of mice. **(F–H)** Median fluorescent intensity was calculated for intracellular cytokine (IFN-g) and markers of T cell cytotoxicity [perforin (PFN) and granzyme B (GZB)] in T effector/effector memory (T_EM_) and T central memory (T_CM_) subsets. All data are derived from n = 5–10 mice per group (at least duplicate observations per sample) and plotted as mean values ± SEM. Significance is presented as described in **Figure 1**. Detailed data (mean values ± SEM and significance) are shown in **Tables S2A** and **S3A**.

The group-specific tSNE plots of the ten metaclusters at the beginning of acute phase (i.e., 10 days pi) are presented in **Figures 2A–D**, and mean values (± SEM) of these T cell subsets are presented in **Figure 2E** and **Table S2A**. These data showed that CD4^−^CD8^−^ double negative (DN) T cells of naïve (P9: 0.76%–3.46%), T_EM_ (P4: 0.25%–0.92%), or T_CM_ (P6: 1.18%–3.12%) phenotypes constituted a small proportion of the total T cells in all the treatment groups. The non-vaccinated/infected (vs. vaccinated/infected) mice exhibited higher levels of premature CD4^+^T_o_ subset (P1) and a decline in the CD4^+^ T naïve (T_N_) subset (P2), while immunized mice exhibited significant increase in CD4^+^T_EM_ subset (P0, nano2/4.*Tc* > p2/4.*Tc*). Regarding CD8^+^T cells, we observed two metaclusters of naive cells, including CD62L^hi^CD44^lo^ (P8) subset that exhibited true naive phenotype and CD62L^intermediate^CD44^low^ (P3) subset that was categorized as activated naïve subset $\left(T_{N}^{L}\right)$. We observed no major changes in the frequencies of the CD8^+^ T cell subsets in any of the infected (vs. non-infected) groups of mice at 10 days pi. At a functional level, the IFN-γ expression was increased in CD4^+^T_EM_ (P0) subset in vaccinated/infected (nano2/4.*Tc* > p2/4.*Tc*) mice (**Figure 2F** **and** **Table S3A**). The PFN production was increased in all of the T_EM_ and T_CM_ subsets in infected mice (nano2/4.*Tc* > p2/4.*Tc* or *Tc* only, **Figure 2G** and **Table S3A**). Except for the GZB-expressing DN and CD8^+^ T_EM_ populations, the maximal increase in GZB expression was also noted in nano2/4.*Tc* mice (vs. p2/4.*Tc* or *Tc* only, **Figure 2H** and **Table S3A**). These results suggest that nano2/4 was most effective in eliciting an early T cell response to *T. cruzi* infection evidenced by **a)** transition of the CD4^+^T cells into effector phenotype, **b)** IFN-γ production by CD4^+^T_EM_ subset; and **c)** production of cytolytic molecules (PFN/GZB) by T cell subsets of T_EM_ and T_CM_ phenotypes in nano2/4*.Tc* (vs. p2/4.*Tc* or *Tc* only) mice.

**Figure 3:**
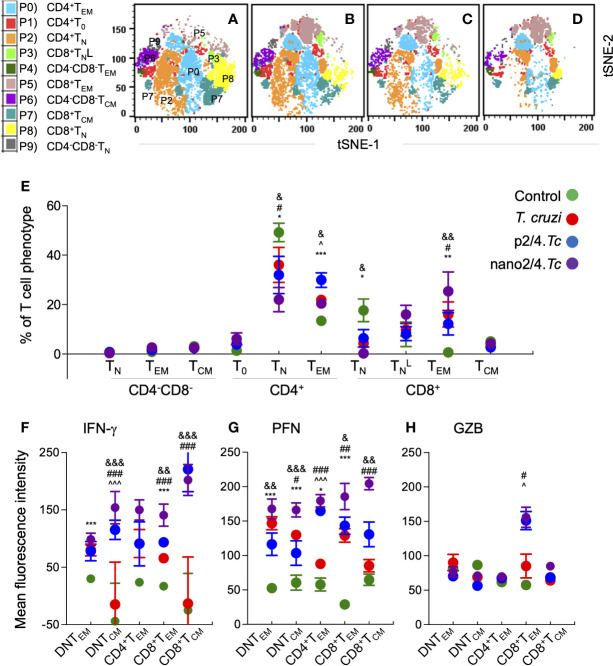
Effect of nanovaccine on dynamics of T cell sub-populations in peak parasitemic phase. C57BL/6 mice were immunized, infected, and euthanized on day 21 pi corresponding to peak parasitemic phase. Single cell suspensions of splenocytes were labeled with fluorescent-conjugated antibodies and analyzed by flow cytometry. FlowSOM analysis of CD3^+^ splenic T cells of mice (*n* ≥ 5 per group) based on the expression levels of CD4, CD8, CD25, CD62L, and CD44 antigens generated self-organizing ten meta-clusters (referred as P0–P9). **(A–D)** The tSNE (t-distributed stochastic neighbor embedding) plots were generated with non-linear reduction method to visualize all the 10 sub-populations in two-dimensional space in control **(A)**, infected **(B)**, p2/4.*Tc* **(C)**, and nano2/4.*Tc* **(D)** groups of mice. **(E)** Bar graph shows P0–P9 sub-populations of T cells in the four groups of mice. **(F-H)** Median fluorescent intensity was calculated for intracellular cytokine (IFN-γ) and markers of T cell cytotoxicity [perforin (PFN) and granzyme B (GZB)] in T central memory (T_CM_) and T effector/effector memory (T_EM_) subsets. All data are derived from n = 5–10 mice per group (≥2 observations per sample) and plotted as mean values ± SEM. Significance is presented as described in **Figure 1**. Detailed data (mean values ± SEM and significance) are shown in **Tables S2B** and **S3B**.

To capture the T cell profile in vaccinated and non-vaccinated mice during parasite replication phase, we defined and visualized the abundance of ten T cell meta-clusters in each group at 21 days pi (**Figures 3A–D** and **Table S2B**). These data showed no major change in the frequencies of DN T subsets (P9, P6, P9) and a decline in the frequencies of CD4^+^ and CD8^+^ naïve T cells (P2 and P8) in all of the infected groups (vs. non-infected/control group) (**Figure 3E**). Further, CD4^+^T_EM_ subset (P0) was highest in p2/4.*Tc* mice while the $CD8^{+}T_{N}^{L}$ and CD8^+^T_EM_ subsets (P3 & P5) were highest in nano2/4.*Tc* mice (**Figure 3E**). At a functional level, we observed significant increase in IFN-γ production by all T_EM_ and T_CM_ subsets in vaccinated/infected mice (nano2/4*.Tc* > p2/4*.Tc*) while only T_EM_ subsets produced IFN-γ in non-vaccinated/infected mice (**Figure 3F** and **Table S3B**). Likewise, PFN production was enhanced in all T_EM_ and T_CM_ subsets of vaccinated/infected mice (nano2/4*.Tc* > p2/4*.Tc*), while fewer T cell subsets produced low levels of PFN in non-vaccinated/infected mice (**Figure 3G** and **Table S3B**). The GZB expression was noticeably enhanced in CD8^+^T_EM_ subset only in vaccinated/infected mice **(** **Figure 3H** and **Table S3B**). These data suggest that without the help of a vaccine, host responds to *T. cruzi* by delayed and sub-par T cell activation and low levels of production of effector molecules. Prophylactic immunization with nano2/4 (more effective than p2/4) enhanced the differentiation of naïve T cells toward CD4^+^ and CD8^+^ T_EM_ and T_CM_ phenotypes and increased the production of IFN-γ that is critical for induction of innate/adaptive immunity and of cytolytic molecules (PFN, GZB) that are required for controlling the intracellular pathogen.

Comparative analysis of T cell profile at 21 days vs. 10 days pi is presented in **Figure S2**. Though present at minimal frequencies, DN T_N_ subset decreased, DN T_EM_ increased and DN T_CM_ subset was not changed in infected mice (**Figure S2A**). Further, CD4^+^T_o_ subset (P1) was decreased in non-vaccinated/infected mice, CD4^+^T_N_ subset (P2) was decreased in vaccinated/infected mice (nano2/4.*Tc* > p2/4.*Tc*), and CD4^+^ T_EM_ population (P0) was increased by 44.9% in p2/4.*Tc* and decreased by 14.5% in nano2/4.*Tc* mice at 21 days (vs. 10 days) pi. With respect to CD8^+^T cells, the CD8^+^T_N_ subset was decreased and the $CD8^{+}T_{N}^{L}$ (178-644%) and CD8^+^T_EM_ (994%–2390%) subsets were increased in all infected groups at 21 days (vs. 10 days) pi, and maximal changes were noted in nano2/4-immunized mice. The CD8^+^T_CM_ subset was increased in non-vaccinated/infected mice only at 21 days (vs. 10 days) pi (**Figure S2A**). When comparing the functional response, the IFN-γ expression level was increased in all of the T_EM_ and T_CM_ subsets in infected mice at 21 days (vs. 10 days) pi, and CD8^+^T_EM_ and T_CM_ subsets exhibited maximal increase in IFN-γ production in nano2/4.*Tc* mice (**Figure S2B**). The PFN production was increased in all the T_EM_ and T_CM_ subsets in vaccinated/infected mice while fewer effector T cell subsets exhibited increase in PFN production in non-vaccinated/infected mice at 21 days (vs. 10 days) pi (**Figure S2C**). In comparison, GZB production was generally decreased in all infected groups, while only CD8^+^T_EM_ subset exhibited increaser in GZB levels in vaccinated/infected groups at 21 days (vs. 10 days) pi (**Figure S2D**). These our results suggest that at the peak of acute infection, maximum proliferation of CD8^+^ T_EM_ takes place in nano2/4-immunized mice, while CD4^+^T_EM_ subset was still expanding in p2/4-immunized mice. The observation of a decline in T cell expansion in nanovaccine immunized mice, when comparing 21 days vs. 10 days pi (**Figure S2A**), indicates that the proliferation time of effector phenotypes of both CD4^+^ and CD8^+^ T cells occurred early on and this vaccine composition reduced the time required for better resolution of infection. Overall, nano2/4 was more effective than p2/4 in signaling activation of CD4^+^ and CD8^+^ T cells at a faster rate, and in stimulating the production of high amounts of the intracellular effector molecules.

### Efficacy of the Nanovaccine Against Repeat Exposure to *T. cruzi*

**Figure 4:**
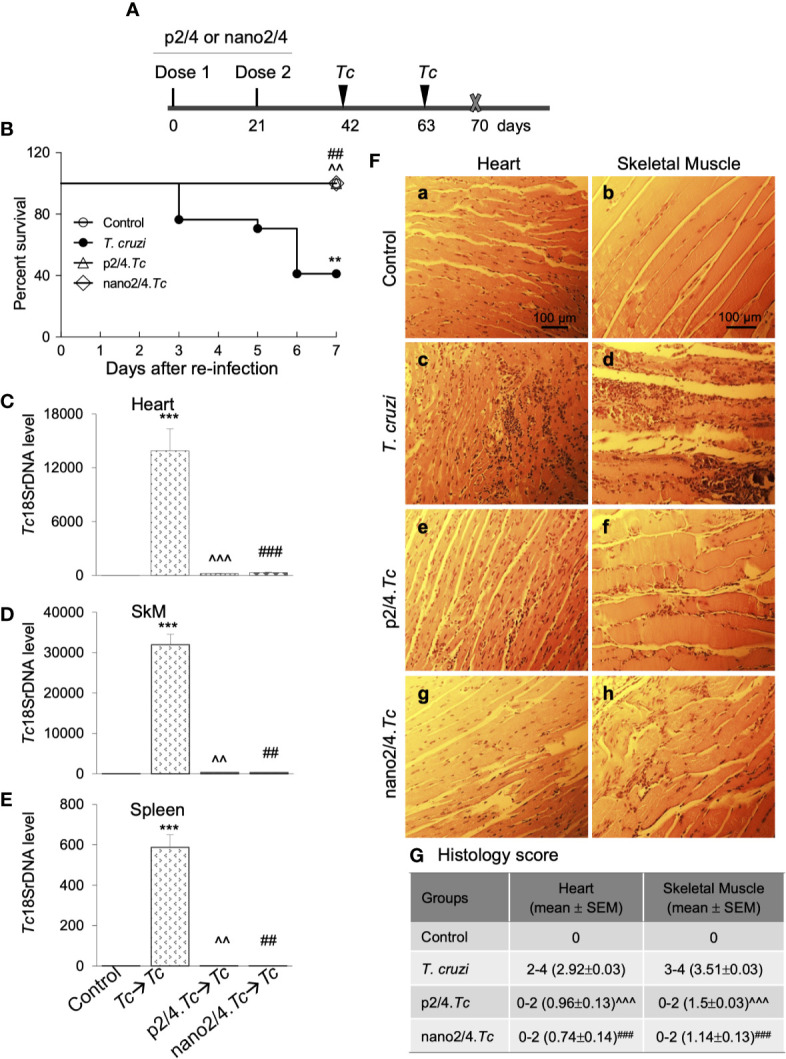
Prophylactic efficacy of nanovaccine against *T. cruzi* re-challenge infection. **(A)** Schematic of experimental design. C57BL/6 mice were vaccinated with p2/4 or nano2/4 on day 0 and day 21 and infected with *T. cruzi* (10,000 trypomastigotes per mouse) on day 42 and day 63. Mice were euthanized on day 70 corresponding to 7 days after 2^nd^ challenge. **(B)** Kaplan Meier survival curve for all groups of mice (*n* ≥ 5 mice per group). **(C**–**E)** Tissue parasite burden. The qPCR evaluation of *Tc18SrDNA* levels in heart **(C)**, skeletal muscle **(D)**, and spleen **(E)** tissues at 7 days after re-challenge was performed as described in Materials and Methods. Bar graphs show mean value ± SEM derived from ≥ 2 observations per sample (*n* = 5 mice/group/experiment). **(F, G) **Tissue inflammatory infiltrate after re-challenge with *T. cruzi*. Paraffin-embedded 5-µm tissue sections were obtained 7 days after re-challenge and examined by H&E staining. Shown in **(F)** are representative images of the heart and skeletal muscle tissue sections from non-vaccinated/non-infected (a, b)****, non-vaccinated/*T. cruzi-*infected (c, d)****, p2/4.*Tc* (e, f)****, and nano2/4.*Tc* (g, h)**** groups of mice (magnification: 20×). Inflammatory score was calculated as described in Materials and Methods, and presented in **(G)**. Significance was calculated by student’s t-test (^*^ no infection vs. *Tc*) and one-way ANOVA/Tukey’s (^^^ *Tc* vs. p2/4.*Tc* and ^#^ *Tc* vs. nano2/4*.Tc*) and one, two, and three symbol characters annotate the p values of <0.05, <0.01, and <0.001, respectively.

To determine if vaccine- and *T. cruzi-*induced immunity is protective against re-infection, we challenged mice 21 days after the 2^nd^ immunization with p2/4 or nano2/4, and then re-challenged with *T. cruzi* 21 days after the first exposure. Mice were monitored daily and surviving mice were euthanized 7 days after 2^nd^ infection to examine tissue pathology (**Figure 4A**).

Upon re-infection with *T. cruzi*, non-vaccinated/infected mice exhibited clinical features of sickness (hunched posture, ruffled fur, lethargic), and 59% (10 out of 17) of non-vaccinated/infected mice succumbed within 7 days after re-infection (**Figure 4B**). The surviving non-vaccinated/re-infected mice exhibited very high levels of tissue parasite burden in heart, skeletal muscle, and splenic tissues that were 2-fold, 4-fold, and 1.5-fold higher than was observed after 1^st^ infection (compare **Figures 4C–E** with **Figures 1B–D**). No mortality was observed in re-infected mice that had received prophylactic p2/4 or nano2/4 vaccine as was also noted in mock-infected/mock-vaccinated control mice (**Figure 4B**). Parasites were almost undetectable in tissues of immunized mice after re-challenge with *T. cruzi*. We noted 44- to 65-fold, 93- to 102-fold, and 501- to 643-fold lower levels of parasite burden in heart, skeletal muscle and splenic tissue of mice given prophylactic p2/4 or nano2/4 compared to what was observed in non-vaccinated/infected mice after re-challenge infection (**Figures 4C–E**). Furthermore, tissue parasite burden in vaccinated mice after re-challenge was even lower than that noted after 1^st^ exposure (compare **Figures 4C–E** with **Figures 1B–D**).

Extensive and large foci of tissue inflammatory infiltrate were observed in heart (histology score: 2.92) and skeletal muscle (histology score: 3.51) tissues of mice at 7 days post re-infection (**Figures 4F.c, d, G**). Tissue inflammatory foci and tissue degeneration were more pronounced after re-infection in non-vaccinated mice (compare **Figures 4F.c, d** with **Figures 1E.c, d**). The p2/4- and nano2/4-immunized mice showed noteworthy decline in the inflammatory infiltrate in the heart (histology score: 0.74-0.96, ^^^ *p* < 0.001) and skeletal muscle (histology score: 1.14–1.5, ^^^ *p* < 0.001) tissue as compared to what was observed in non-vaccinated/infected mice (**Figures 4F, compare c and d with e–h, 4G**). Together, these results suggest that a) *T. cruzi*-induced immunity offered no protection from repeat exposure to the same pathogen isolate in this murine model; b) TcG2- and TcG4-based vaccines offer significantly better control of tissue parasite burden after re-infection than was observed after 1^st^ infection; and c) vaccine-induced parasite control was associated with pronounced decline in tissue inflammatory pathology and tissue injuries.

### T Cell Profile in Response to Repeat *T. cruzi* Infection (± Nanovaccine)

**Figure 5:**
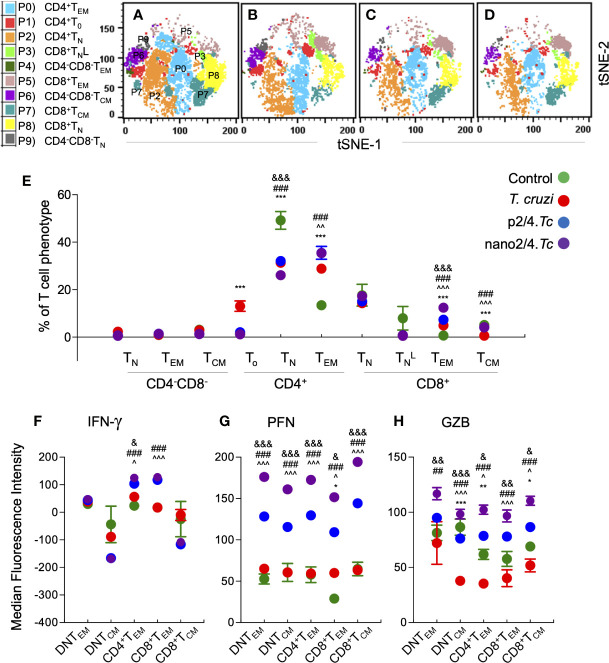
Effect of nanovaccine on dynamics of functional activation of T cell sub-populations after re-exposure to *T. cruzi*. C57BL/6 mice were immunized with two doses of p2/4 or nano2/4 at 21 days interval, infected on day 21 after 2^nd^ vaccine dose, re-challenged on day 21 after first infection, and euthanized on day 7 after re-challenge. Single cell suspensions of splenocytes were labeled with fluorescent-conjugated antibodies and analyzed by flow cytometry. FlowSOM analysis of CD3^+^ splenic T cells of mice (1 × 10^5^ live cells per mouse, *n* ≥ 5 per group) based on the expression levels of CD4, CD8, CD25, CD62L, and CD44 antigens generated self-organizing ten meta-clusters (referred as P0–P9). **(A–D)** The tSNE (t-distributed stochastic neighbor embedding) plots were generated with non-linear reduction method to visualize all the 10 sub-populations in two-dimensional space in non-vaccinated/non-infected **(A)**, non-vaccinated/infected **(B)**, p2/4.*Tc* **(C),** and nano2/4.*Tc* **(D)** groups of mice. **(E)** Bar graphs show P0–P9 sub-populations of T cells in four groups of mice. **(F–H)** Median fluorescent intensity was calculated for intracellular cytokine (IFN-γ) and markers of T cell cytotoxicity [perforin (PFN) and granzyme B (GZB)] in T central memory (T_CM_) and T effector/effector memory (T_EM_) subsets. All data are derived from *n* ≥ 5 mice per group (≥ 2 observations per sample) and plotted as mean values ± SEM. Significance is presented as described in **Figure 1**. Detailed data (mean values ± SEM and significance) are shown in **Tables S2C** and **S3C**.

As above, we first defined and visualized the abundance of 10 meta-clusters of T cells in vaccinated and non-vaccinated mice at 7 days post reinfection by t-SNE plots (**Figures 5A–D**). The mean values (± SEM) of the T cell subsets are presented in **Figure 5E** and **Table S2C**, and functional profile of the T cell subsets are presented in **Figures 5F–H** **and** **Table S3C**. Comparative analysis of T cell profile at 7 days after re-infection vs. 21 days post 1^st^ infection is presented in **Figure S3**.

Upon re-challenge, mice reverted to T cell effector phenotypes as was observed at early stage of 1^st^ infection (i.e., 10 days pi in **Figure 2**). The DN T subsets (P9, P4, and P6) largely remained non-responsive and CD8^+^ naïve subset (P3) contracted in all infected (vs. control) groups, and CD4^+^T_o_ were primarily enhanced in non-vaccinated/infected mice (**Figure 5E** **and** **Table S2C**). The CD4^+^T_EM_ subset (P0) was increased in all infected (vs. control) groups (p2/4.*Tc* = nano2/4.*Tc* > *Tc* only, **Figure 5E** **and** **Table S2C**), and maximal percentage increase in CD4^+^T_EM_ subset at 7 days after re-challenge (vs. 21 days after 1^st^ infection) was observed in nano2/4.*Tc* mice (**Figure S3A**). The CD8^+^T_EM_ subset (P5), though in contraction phase when compared to that observed at 21 days after 1^st^ infection (**Figure S3A**), remained significantly higher in nano2/4.*Tc* mice compared to other groups of infected mice (**Figure 5E**). Functional analysis of T cell subsets after reinfection showed that the IFN-γ production was practically absent in DN T cell subsets and CD8^+^T_CM_ subpopulation, and maintained at higher levels in CD4^+^T_EM_ and CD8^+^T_EM_ subsets in vaccinated (nano2/4.*Tc*> p2/4.*Tc*) mice but not in non-vaccinated/infected mice (**Figure 5F**). Overall intensities of PFN and GZB in all of the T_EM_ and T_CM_ subsets were very high after re-infection in vaccinated mice (nano2/4.*Tc* > p2/4.*Tc*) while non-vaccinated/infected mice continued to produce none-to-low levels of PFN/GZB after reinfection (**Figures 5G, H**). When comparing the effector molecule levels after re-challenge with that observed at 21 days post 1^st^ infection, the intensity of IFN-γ production was increased or maintained at high levels in CD4^+^ and CD8^+^ T_EM_ subsets (P0 & P5) in vaccinated groups (**Figure S3B**). The PFN expression was maintained at high levels in all T_EM_ and T_CM_ subsets in vaccinated/infected mice. With respect to GZB production, while CD8^+^T_EM_ (P5) were contracting, CD4^+^T_EM_ and CD8^+^T_CM_ (P0 & P7) subsets exhibited increased GZB expression in vaccinated/infected mice (**Figure S3D**). All the effector molecules exhibited a downward trend in non-vaccinated/infected mice after re-challenge (**Figures S3B–D**). Overall, these results suggest that upon re-challenge, vaccinated mice were able to enhance or maintain the CD4^+^ and CD8^+^ T_EM_ sub-populations that produced high levels of effector molecules (nano2/4>p2/4). In comparison, non-vaccinated/infected mice responded to re-challenge with no increase in effector T cell populations and an overall decline in their production of effector/cytotoxic molecules.

### Plasma Cytokines as Biomarkers of Host Immunity to Infection and Reinfection (± Nanovaccine)

**Figure 6:**
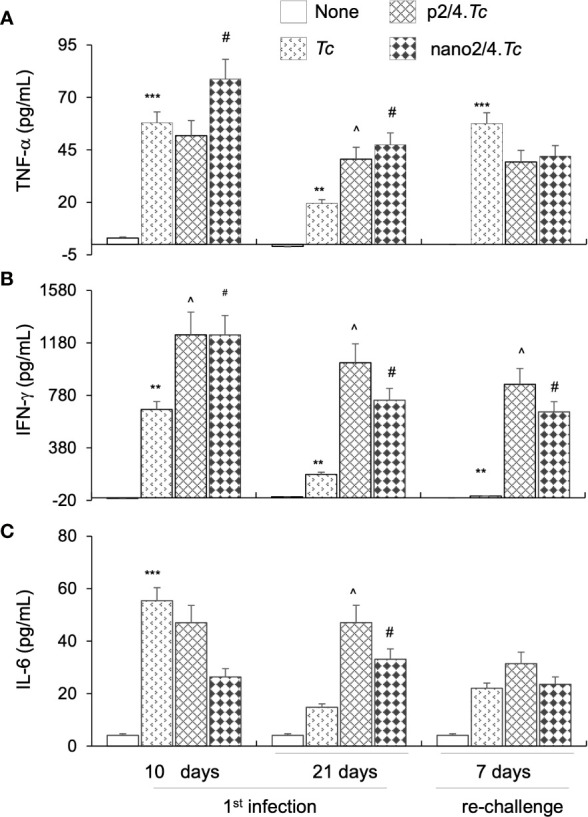
Systemic markers of inflammation in mice after acute infection and re-challenge with *T. cruzi* (± nanovaccine). Mice were immunized and infected and re-challenged as described in **Figures 1A** and **4A**. Serum samples were obtained at day 10 and 21 post-infection and day 7 after re-challenge, and TNF-α **(A)**, IFN-g **(B)**, and IL-6 **(C)** levels were evaluated by using commercially available ELISA kits. Data (mean ± SEM) are representative of triplicate observations per sample (*n* ≥ 5 mice per group). Significance was calculated by student’s t-test (* no infection vs. *Tc*) and one-way ANOVA/Tukey’s (^^^ *Tc* vs. p2/4.*Tc* and ^#^ *Tc* vs. nano2/4*.Tc*). One, two, and three symbol characters were used to annotate the p values of <0.05, <0.01, and <0.001, respectively.

Finally, we measured plasma levels of TNF-α, IFN-γ, IL-6, and IL-1β cytokines by an ELISA to determine if systemic secretion of cytokines offers an indication of anti-parasite protective immunity after infection and re-infection. We noted a potent increase in plasma levels of inflammatory cytokines in all infected groups of mice. The p2/4- and nano2/4-immunized mice elicited high plasma levels of TNF-α and IFN-γ at 10 days pi that were maintained in high range at 21 days pi and at 7 days after re-infection (**Figures 6A–C**). The TNF-α levels in non-vaccinated/infected mice were comparable to vaccinated/infected mice at 10 days pi, lowest at 21 days pi and spiked again in response to re-infection. Specifically, plasma IFN-γ levels were increased by 1.84-fold, 4.3- to 5.7-fold, and >50-fold in vaccinated/infected (vs. non-vaccinated/infected) mice at day 10 and day 21 pi and 7 days after re-infection, respectively (^p < 0.05, **Figures 6A–C**). The plasma levels of IFN-γ in non-vaccinated/infected were significantly induced, albeit at lower levels than in vaccinated/infected mice, at 10 days pi and after that very low levels of IFN-γ were detectable during the acute parasitemic phase (i.e., 21 days pi) as well as after re-infection in non-vaccinated/infected mice (**Figures 6A–C**). The increase in TNF-α + IFN-γ was maximal in vaccinated mice (nano2/4.*Tc* ≥ p2/4.*Tc* > *Tc* only) and correlated with the control of parasite burden at all stages of infection and re-infection. The IL-6 levels were higher in p2/4.*Tc* group as compared to that noted in nano2/4.*Tc* group at all stages of infection and reinfection. In non-vaccinated/infected mice, IL-6 levels were high at 10 days pi and then it remained at low levels at 21 days pi and 7 days after reinfection. No detectable increase in IL-1β was observed in plasma of any of the infected groups of mice (data not shown). These results, along with the parasite burden data presented in **Figures 1** and **4**, suggest that while *T. cruzi* infection induces systemic cytokine response, it is not maintained and fails to control parasite dissemination and replication as well as fails to respond to and provide protection from re-infection. Immunization with p2/4 and nano2/4 elicited sustained TNF-α– and IFN-g–dominated systemic immunity that correlated with control of parasite dissemination and replication as well as protection from repeat infections.

## Discussion

Chagas disease is a multisystemic parasitic disease which affects ~8 million people worldwide. Two drugs, benznidazole and nifurtimox, are recommended for the anti-parasitic treatment of children (<14 years old) and adults who might be acutely infected with *T. cruzi* (28, 29). Some studies recommend that seropositive adults who are in indeterminate-to-chronic phase with mild-to-no symptoms of heart disease should also be treated with benznidazole to prevent advanced disease progression (30, 31). Yet, many adults do not complete the therapeutic regimen due to adverse events that can also contribute to drug resistance in *T. cruzi* (32), and these drugs are contraindicated for the treatment of pregnant women (33) and Chagas patients with clinical manifestations of cardiac disease (34–36). Further, *T. cruzi* isolates from heterogenous background are not equally susceptible to these drugs (37, 38). It is, thus, believed by the research community that an overall increase in access to anti-parasite therapies will limit the disease progression in infected individuals and vaccines are needed to prevent the spread of *T. cruzi* and Chagas disease (39, 40). Historically, pathogen-based attenuated vaccines (e.g., smallpox, plague, and measles) are shown to elicit robust immunity and provide protection from repeat infections (41–44). However, our findings in this study indicate that primary *T. cruzi* infection elicits delayed, sub-par T cell immunity that fails to provide protection against re-infection. Thus, vaccines made of killed or live-attenuated parasite are likely to offer limited-to-no protection from *T. cruzi*, as has also been documented in some studies (45). In this context, a subunit vaccine is perhaps the best choice for control of *T. cruzi* infection.

Previously, we have employed unbiased computational/bioinformatic algorithm to select *T. cruzi* antigens and tested their potential as vaccine candidates in mice. Of the eleven candidates identified, TcG2 and TcG4 were most promising based on several selection features (9, 10). These two candidates were cloned in a eukaryotic expression plasmid (pCDNA3.1) and tested as an experimental vaccine in mice and dogs to provide proof-of-principle for their efficacy in controlling *T. cruzi* infection (12, 13, 46, 47). However, pCDNA3.1 is not permissible for use in humans due to regulatory issues such as potential integration in human chromosome and presence of antibiotic selective marker. The TcG2 and TcG4 antigens consist human major histocompatibility complex class I and class II epitopes thus rationalizing their further testing in a human permissible expression plasmid. Thus, we sub-cloned the vaccine candidates in a nanoplasmid designed in accordance with the FDA regulatory guidance and tested its prophylactic efficacy in mice in this study.

In general, T cell response can be divided into three phases, i.e., early priming and expansion, resolution and contraction, and development of memory response. During the priming phase, T cells differentiate into effector cells and produce cytotoxic and cytolytic effector molecules. After clearance of the intracellular organism, majority of the effector T cells die in the contraction phase and rest are maintained as memory T cells for long-term protection against re-exposure to the pathogen (48). We noted almost no expansion of T cells at early time point of acute infection that is also verified by others (49, 50). Our current findings and published reviews (1, 51) also indicate that adaptive T cell immunity is delayed and peaks only by 3 to 4 weeks after initial infection, which allows unhindered dissemination and replication of the parasite in various cells and tissues (52, 53). Furthermore, we noted exhaustion of T cell immunity and severe tissue pathology in non-vaccinated/infected mice upon re-exposure to the pathogen. These findings are in alignment with literature (54) and allow us to surmise that a lack of early T cell expansion and function and their inability to maintain memory response or enhance effector response contributes to susceptibility to infection and reinfection to *T. cruzi* in endemic areas.

The CD4^+^, CD8^+^, and CD4^−^CD8^−^ DN T cells have the highest ability to produce IFN-g that rapidly augments T cell proliferation by autocrine and paracrine signaling (55). Studies testing the efficacy of a variety of subunit vaccines have indicated that both CD4^+^ and CD8^+^ T cells are vital for providing protection from infective and intracellular *T. cruzi*; however, little is known about the phenotype of the T cells that must be activated to control *Tc* infection. The DN αβ T cells producing IFN-g correlated with severe form of CD and DN γδ T cells producing IL-10 correlated with less severe form of CD in humans (56). However, the role of DN T cells, if any, in acute *T. cruzi* infection is not known. In this study, we have identified ten T cell metaclusters in the splenic environment of *T. cruzi* infected mice, and shown that CD4^+^T_EM_, CD8^+^T_EM_, and CD8^+^T_CM_ subsets producing IFN-g and PFN/GZB were maximally expanded in nano2/4-immunized mice, and are likely of most significance for controlling acute *T. cruzi* infection. This is because IFN-g/TNF-α, and CD8^+^ T cells and to some extent CD4^+^ T cells secreting pore forming PFN and serine protease GRZ play a major role in killing of intracellular pathogens (57–59). Our findings of an increase in PFN/GZB production by DN cells of T_EM_ and T_CM_ phenotype in nano2/4 vaccinated mice in response to infection and reinfection and of IFN-g production during parasitemic phase at 21 days pi suggest that DN T cells, though present at low frequency, may play a role in providing resistance to acute *T. cruzi* infection. However, this needs to be verified in future studies with depletion of DN T cells in experimental models of *T. cruzi* infection.

We recorded fewer parasites in heart, skeletal muscle and spleen tissue of nano2/4-immunized mice as compared to p2/4-immunized mice, thus indicating that nano2/4 offers improved protection against *T. cruzi* infection. Comparing the systemic T cell profile, we observed that the CD4^+^T_EM_ subset was maximally activated in p2/4-immunized mice while CD8^+^T_EM_ subpopulation peaked in nano2/4-immunized mice during the acute parasitemic phase. We postulate that CD4^+^T_EM_ subpopulation secreting more IFN-g at an early time-point helped the proliferation and functional activation of CD8^+^ T_EM_ subset by 21 days pi and dominance of CD8^+^ T_EM_ cells was useful in final clearance of intracellular parasite in nano2/4-immunized/infected mice. It is also worth noting that nano2/4 vaccination resulted in fewer infiltration of immune cells in the myocardial and skeletal muscle of infected mice. Therapeutic delivery of nano2/4 also reduced the myocardial infiltration of inflammatory cells that drives the development of cardiac fibrosis and Chagas cardiomyopathy (18). These findings indicate that modified nano backbone vehicle carrying TcG2 and TcG4 antigens protected the infected host more efficiently than was observed with pCDNA3.1-based antigen delivery in this study and previously published reports (12, 13, 46, 47).

We also assessed the efficacy of vaccine at another level by evaluating mortality as an outcome after re-infection at the peak acute phase of primary infection. We observed no death in vaccinated mice, whereas non-vaccinated mice exhibited >50% mortality associated with degeneration of organs due to high parasitemic burden after re-infection. The expansion of CD4^+^T_EM_ subset was noted in all groups of mice after infection, but we cannot ignore the cytotoxic and cytolytic effector molecules that were maximally enhanced in nano2/4-vaccinated mice after re-infection. This could likely be because CD8^+^ T_EM_ proliferation as well as their production of effector molecules was increased at 21 days of primary infection, and this subset may not have contracted in nano2/4-immunized mice (vs. other groups) at reinfection.

In summary, we have demonstrated improved efficacy of nano2/4 in signaling polyfunctional DN, CD4^+^ and CD8^+^ effector T cell responses capable of providing protection against *T. cruzi* infection. We showed that nano2/4 enhanced the production of cytotoxic intracellular molecules by the existing T cells immediately after infection that was followed by rapid proliferation and further functional activation of CD4^+^ and CD8^+^ effector T cells, and it had the ability to keep the parasite dissemination and replication at a minimal level. Furthermore, nano2/4 vaccination shortened the time-course of development and early activation of most efficient CD8^+^ T_EM_ cells for elimination of intracellular parasites. Most importantly, nano2/4-dependent quick, recall response prevented the severe increase in tissue parasite burden and injurious pathology that otherwise was observed in non-vaccinated mice after re-exposure to pathogen.

A limitation of this study is that vaccine protection was primarily examined against primary and re-challenge infection with the same parasite isolate, while insect vector carrying heterogenous parasites might circulate in the endemic areas, and, therefore, a host may encounter multiple parasite isolates during repeat infections. Further, this study focused on evaluating the vaccine efficacy against acute parasitemia, while clinical phase of *T. cruzi* infection is presented with development of Chagas cardiomyopathy. To address these limitations, we will aim to evaluate in future studies the vaccine efficacy against re-infection with diverse parasite isolates as well as against chronic phase of parasite persistence and Chagas cardiomyopathy and utilize advanced panel of antibodies for deeper analysis of T cell phenotypes.

## Data Availability Statement

The original contributions presented in the study are included in the article/**Supplementary Materials**. Further inquiries can be directed to the corresponding author.

## Ethics Statement

All animal experiments were conducted following the National Institutes of Health guidelines for housing and care of laboratory animals and in accordance with protocols approved by the Institutional Animal Care and Use Committee (protocol number 08-05-029) at The University of Texas Medical Branch at Galveston. All experiments were conducted in ABSL2/BSL2-approved laboratory and all personnel have received appropriate ABSL2/BSL2 training.

## Author Contributions

NG provided financial support and conceived the study. NL and IC designed and performed the experiments, analyzed the data, and confirmed the accuracy of their data presentation in the manuscript. NG and IC wrote and edited the manuscript. All authors contributed to the article and approved the submitted version.

## Funding

This work was supported in part by a grant from the National Institutes of Health/National Institute of Allergy and Infectious Diseases (R01AI136031) to NG.

## Conflict of Interest

The authors declare that the research was conducted in the absence of any commercial or financial relationships that could be construed as a potential conflict of interest.

## Supplementary Material

Supplementary Figure 1 Schematics for the analysis of T-cell population profile. Mice were immunized with p2/4 or nano2/4, infected with *T. cruzi*, and euthanized at day 10 and day 21 after challenge and at day 7 after re-infection, as described in **Figures 1A** and **2A**. Splenic cells from all mice (*n* ≥ 5 per group) were analyzed by flow cytometry. CD3^+^ splenic T cells (1 × 10^5^ live cells per mouse) were cumulatively analyzed by FlowSOM software. **(A)** CD3^+^T cells are presented in a total of 49 nodes in a 7 × 7 grid, based on the expression levels of CD4, CD8, CD25, CD62L, and CD44 antigens. **(B)** Phenotypically similar nodes from panel A were clustered into metaclusters, numbered P0–P9. **(C)** Relative median fluorescent intensities of surface receptors from all the cohorts at all the time points were utilized to visualize as heatmap for 10 meta-clusters of CD3^+^ live T cells. Each row represents a sub-population based on expression of markers presented in the columns. The percentage of cells expressing surface markers is visualized with color scale from green (0) to red (1,000). **(D)** Mean value (± SEM) for the expression levels of the five surface receptors in P0–P9 sub-populations are shown. **(E)** Common t-Distributed Stochastic Neighbor Embedding (t-SNE) presentation of metaclusters.

Supplementary Figure 2 Comparison of dynamics of T cell sub-populations at early and late stages of acute *T. cruzi* infection in vaccinated and non-vaccinated mice. C57BL/6 mice were immunized, infected, and euthanized on day 10 and day 21 pi, as described in **Figure 1A**. Splenocytes were labeled with fluorescent-conjugated antibodies and analyzed by flow cytometry. FlowSOM analysis of CD3^+^ splenic T cells of mice based on the expression levels of CD4, CD8, CD25, CD62L, and CD44 antigens generated self-organizing ten meta-clusters (referred as P0–P9). **(A)** Mean percentages of T cell sub-populations in control, infected, p2/4.*Tc* and nano2/4.*Tc* groups of mice at day 10 and day 21 pi are presented as bar diagram. **(B–D)** Median fluorescent intensity was calculated for IFN-γ, perforin (PFN) and granzyme B (GZB) in T effector/effector memory (T_EM_) and T central memory (T_CM_) subsets. All data are derived from *n* ≥ 5 mice per group (at least duplicate observations per sample). Mean values ± SEM and ^$^ p value <0.05 (day 10 vs. day 21) are presented in all panels.

Supplementary Figure 3 Effect of re-challenge on dynamics of T cell sub-populations in acutely infected mice (± vaccine). C57BL/6 mice were immunized with two doses of p2/4 or nano2/4 at 21 days interval, infected on day 21 after 2^nd^ vaccine dose, re-challenged on day 21 after first infection, and euthanized on day 7 after re-challenge, as described in **Figure 4A**. Splenocytes were labeled with fluorescent-conjugated antibodies and analyzed by flow cytometry. FlowSOM analysis of CD3^+^ splenic T cells of mice based on the expression levels of CD4, CD8, CD25, CD62L, and CD44 antigens generated self-organizing ten meta-clusters (referred as P0–P9). **(A)** Mean percentages of P0–P9 sub-populations of T cells in control, *T. cruzi* only, p2/4.*Tc* and nano2/4*.Tc* groups of mice at 21 days after 1^st^ infection and 7 days after re-challenge are shown. **(B–D)** Median fluorescent intensity was calculated for IFN-γ, perforin (PFN) and granzyme B (GZB) in T effector/effector memory (T_EM_) and T central memory (T_CM_) subsets. All data are derived from *n* ≥ 5 mice per group (at least duplicate observations per sample). Mean values ± SEM and ^$^ p values <0.05 (day 21 post-infection vs. day 7 after re-challenge) are presented in all panels.
