# Supplementary figures and images for: Experimental Nanovaccine Offers Protection Against Repeat Exposures to Trypanosoma cruzi Through Activation of Polyfunctional T Cell Response
Source: Front Immunol. 2020 Dec 22;11:595039. doi: 10.3389/fimmu.2020.595039 (PMC7783422; doi:10.3389/fimmu.2020.595039)

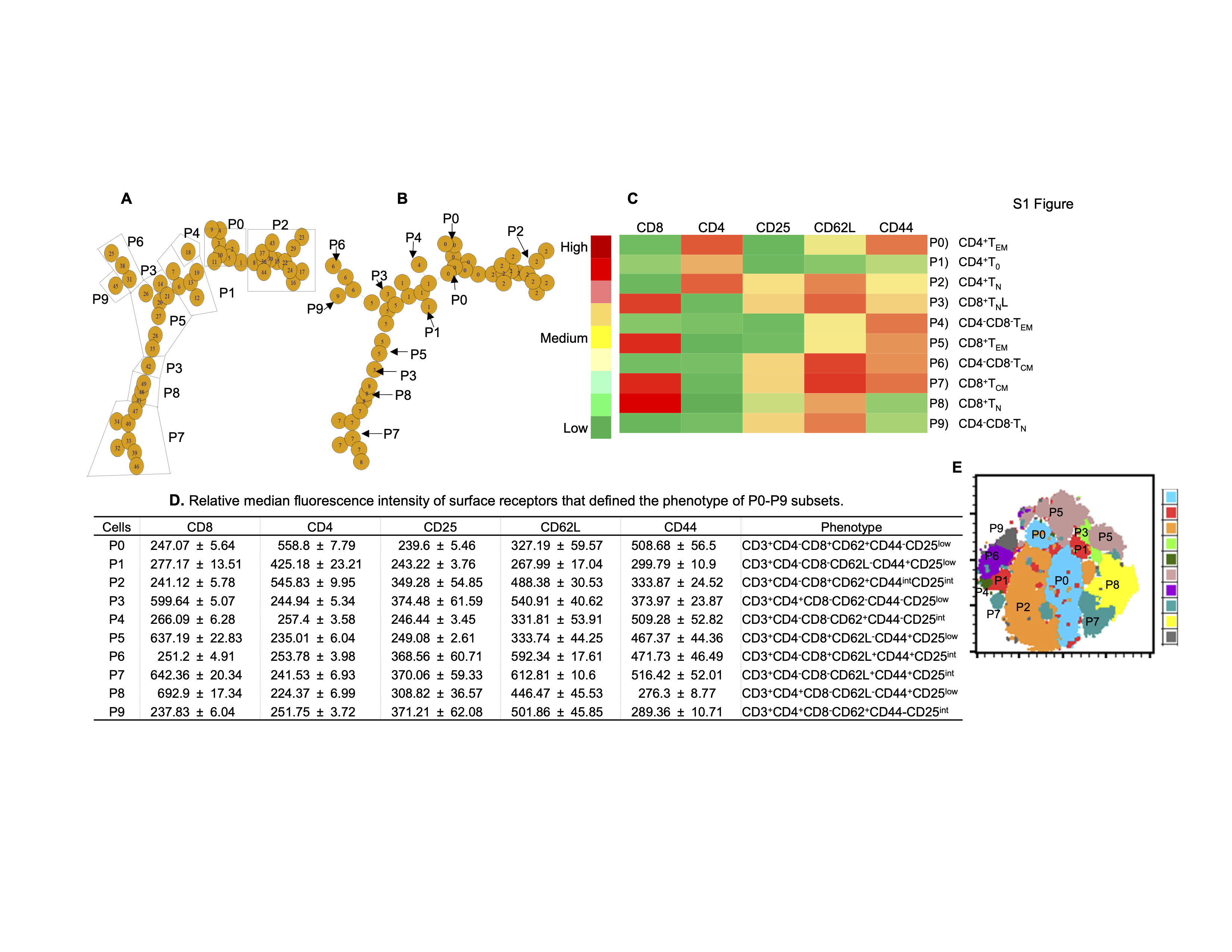

Supplement: Supplementary Figure 1 — Schematics for the analysis of T-cell population profile. Mice were immunized with p2/4 or nano2/4, infected with T. cruzi, and euthanized at day 10 and day 21 after challenge and at day 7 after re-infection, as described in Figures 1A and 2A . Splenic cells from all mice (n ≥ 5 per group) were analyzed by flow cytometry. CD3+ splenic T cells (1 × 10^5^ live cells per mouse) were cumulatively analyzed by FlowSOM software. (A) CD3+T cells are presented in a total of 49 nodes in a 7 × 7 grid, based on the expression levels of CD4, CD8, CD25, CD62L, and CD44 antigens. (B) Phenotypically similar nodes from panel A were clustered into metaclusters, numbered P0–P9. (C) Relative median fluorescent intensities of surface receptors from all the cohorts at all the time points were utilized to visualize as heatmap for 10 meta-clusters of CD3+ live T cells. Each row represents a sub-population based on expression of markers presented in the columns. The percentage of cells expressing surface markers is visualized with color scale from green (0) to red (1,000). (D) Mean value (± SEM) for the expression levels of the five surface receptors in P0–P9 sub-populations are shown. (E) Common t-Distributed Stochastic Neighbor Embedding (t-SNE) presentation of metaclusters. [file Image_1.tiff]

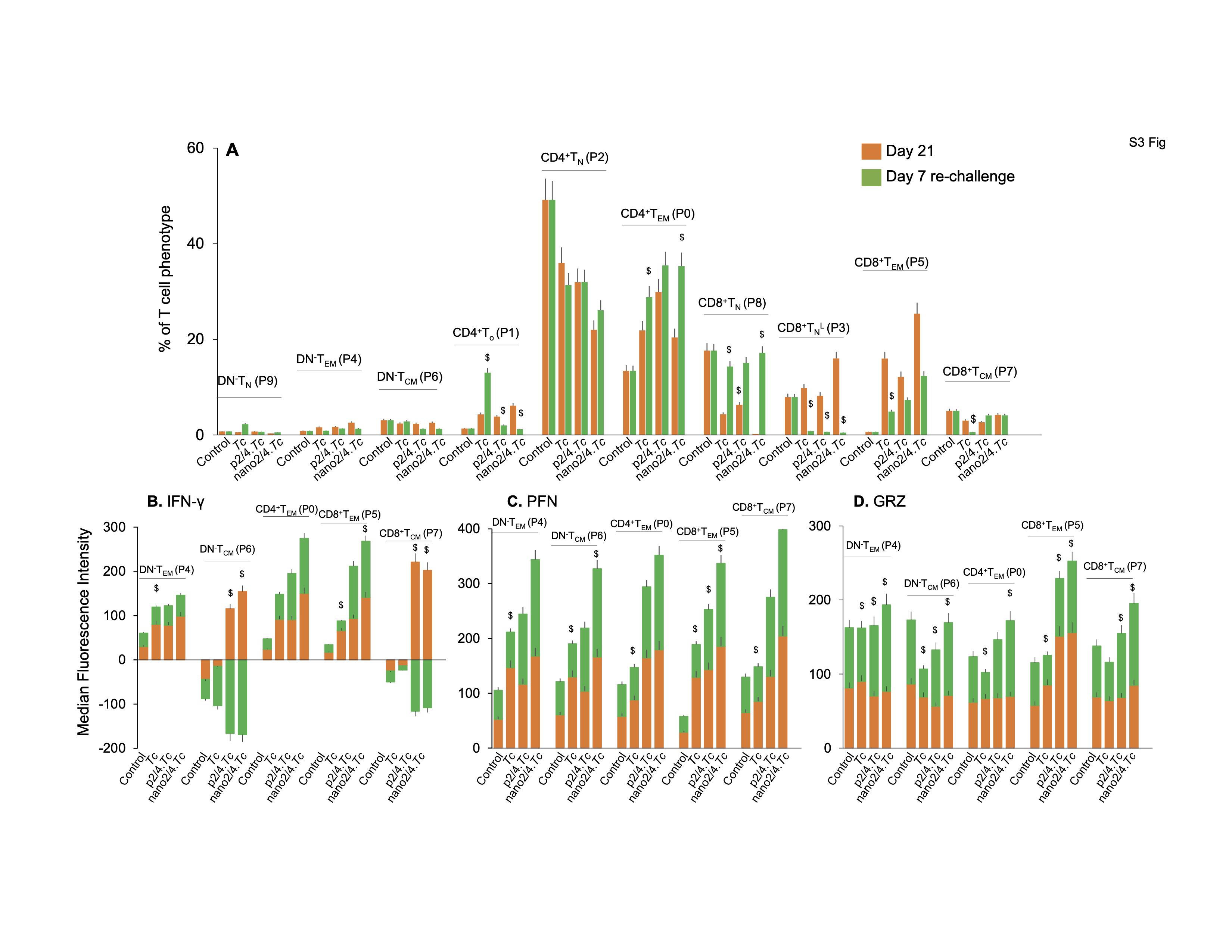

Supplement: Supplementary Figure 2 — Comparison of dynamics of T cell sub-populations at early and late stages of acute T. cruzi infection in vaccinated and non-vaccinated mice. C57BL/6 mice were immunized, infected, and euthanized on day 10 and day 21 pi, as described in Figure 1A . Splenocytes were labeled with fluorescent-conjugated antibodies and analyzed by flow cytometry. FlowSOM analysis of CD3+ splenic T cells of mice based on the expression levels of CD4, CD8, CD25, CD62L, and CD44 antigens generated self-organizing ten meta-clusters (referred as P0–P9). (A) Mean percentages of T cell sub-populations in control, infected, p2/4.Tc and nano2/4.Tc groups of mice at day 10 and day 21 pi are presented as bar diagram. (B–D) Median fluorescent intensity was calculated for IFN-γ, perforin (PFN) and granzyme B (GZB) in T effector/effector memory (TEM) and T central memory (TCM) subsets. All data are derived from n ≥ 5 mice per group (at least duplicate observations per sample). Mean values ± SEM and $ p value <0.05 (day 10 vs. day 21) are presented in all panels. [file Image_2.tiff]

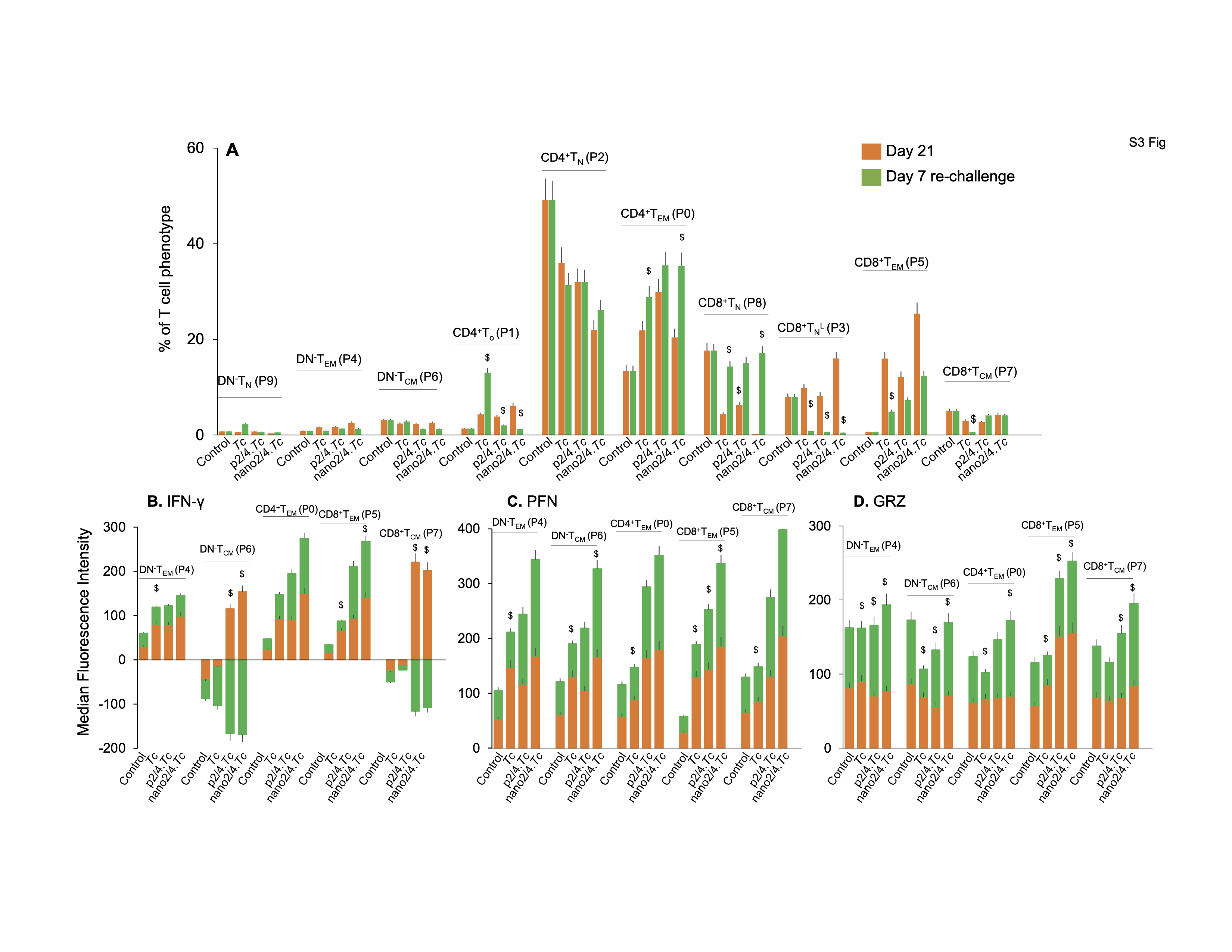

Supplement: Supplementary Figure 3 — Effect of re-challenge on dynamics of T cell sub-populations in acutely infected mice (± vaccine). C57BL/6 mice were immunized with two doses of p2/4 or nano2/4 at 21 days interval, infected on day 21 after 2nd vaccine dose, re-challenged on day 21 after first infection, and euthanized on day 7 after re-challenge, as described in Figure 4A . Splenocytes were labeled with fluorescent-conjugated antibodies and analyzed by flow cytometry. FlowSOM analysis of CD3+ splenic T cells of mice based on the expression levels of CD4, CD8, CD25, CD62L, and CD44 antigens generated self-organizing ten meta-clusters (referred as P0–P9). (A) Mean percentages of P0–P9 sub-populations of T cells in control, T. cruzi only, p2/4.Tc and nano2/4.Tc groups of mice at 21 days after 1st infection and 7 days after re-challenge are shown. (B–D) Median fluorescent intensity was calculated for IFN-γ, perforin (PFN) and granzyme B (GZB) in T effector/effector memory (TEM) and T central memory (TCM) subsets. All data are derived from n ≥ 5 mice per group (at least duplicate observations per sample). Mean values ± SEM and $ p values <0.05 (day 21 post-infection vs. day 7 after re-challenge) are presented in all panels. [file Image_3.tiff]
