# Supplementary material for: Experimental Nanovaccine Offers Protection Against Repeat Exposures to Trypanosoma cruzi Through Activation of Polyfunctional T Cell Response
Source: Front Immunol. 2020 Dec 22;11:595039. doi: 10.3389/fimmu.2020.595039 (PMC7783422; doi:10.3389/fimmu.2020.595039)
Supplement: Supplementary file 4 [file Table_1.docx]

| **S1 Table. Antibodies used for flow cytometry analysis of T cells** | | | | | |
| --- | --- | --- | --- | --- | --- |
| **Marker** | **Color** | **Ex/Em (nm)** | **Antibody cat #** | **Source** |  |
| CD3 epsilon | APC | 640/670 ± 15 | Hamster α m / IgG1, κ (145-2C11) | BD Biosc (553066) |  |
| CD4 | BV510 | 405 / 525 ± 25 | Rat α m / IgG2a, κ (RM4-5) | BD Biosc (563106) |  |
| CD8 | BUV395 | 355/450 ± 25 | Rat α m / IgG2a, κ (53-6.7) | BD Biosc (563786) |  |
| Granzyme B | PE | 561/582 ± 7.5 | Rat α m / IgG2a, κ (NGZB) | eBiosc (12-8898-82) |  |
| IFN-γ | BV711 | 405/710 ± 25 | Rat α m / IgG1, κ (XMG1.2) | BD Biosc (564336) |  |
| Perforin | FITC | 488/530 ± 15 | Rat α m / IgG2a, κ (eBioOMAK-D) | ThermoFisher (11-9392-82) |  |
| CD62L | BV650 | 405/670 ± 15 | Rat α m/ IgG2a, κ (MEL-14) | BD Biosc (564108) |  |
| CD44 | BV786 | 405/780 ± 30 | Rat α m / IgG2b, κ (IM7) | BD Biosc (563736) |  |
| CD25 | BV421 | 405/450 ± 25 | Rat α m / IgM, κ (7D4) | BD Biosc (564571) |  |
| Fixable viability stain 780 | | 759/780 |  | BD Biosci (565388) |  |

| **S2 Table.** Percent positive T cell populations in *T. cruzi* infected mice (± nanovaccine) at 10 days **(A)** and 21 days **(B)** post-infection, and at 7 days after re-challenge infection **(C)**. | | | | |
| --- | --- | --- | --- | --- |
| **Populations** | **Control ^a^** | ***T. cruzi*** | **p2/4.*Tc*** | **nano2/4.*Tc*** |
| **A. Day 10 post-infectio**n | | | | |
| CD4^-^CD8^-^T_N_ (P9) | 0.76±0.09 | 3.46±0.28^***^ | 1.97±0.36^^^^ | 1.55±0.14^###^ |
| CD4^-^CD8^-^T_EM_ (P4) | 0.86±0.15 | 0.25±0.09^**^ | 0.76±0.07^^^^ | 0.92±0.10^###^ |
| CD4^-^CD8^-^T_CM_ (P6) | 3.12±0.37 | 1.18±0.10^***^ | 2.22±0.15^^^^^ | 2.35±0.17^###^ |
| CD4^+^T_0_ (P1) | 1.37±0.36 | 21.66±2.61^***^ | 5.33±0.80^^^^^ | 4.54±0.33^###^ |
| CD4^+^T_N_ (P2) | 49.20±3.74 | 31.32±1.15^***^ | 46.81±1.07^^^^^ | 43.93±1.83^##^ |
| CD4^+^T_EM_ (P0) | 13.43±0.93 | 23.62±2.61^**^ | 20.63±1.63 | 23.90±1.65 |
| CD8^+^T_N_ (P8) | 17.65±4.57 | 14.05±0.68 | 16.48±1.11 | 17.00±0.69^&^ |
| CD8^+^T_N_L (P3) | 7.94±4.98 | 2.81±0.54 | 2.96±0.60 | 2.15±0.28 |
| CD8^+^T_EM_ (P5) | 0.65±0.09 | 1.46±0.12^***^ | 0.89±0.07^^^^^ | 1.02±0.09^#^ |
| CD8^+^T_CM_ (P7) | 5.06±0.9 | 0.21±0.04^***^ | 1.93±0.38^^^^^ | 2.64±0.31^###^ |
| **B. Day 21 post-infection** | | | | |
| CD4^-^CD8^-^T_N_ (P9) |  | 0.59±0.10 | 0.74±0.20 | 0.33±0.03^&^ |
| CD4^-^CD8^-^T_EM_ (P4) |  | 1.63±0.30^*^ | 1.70±0.41 | 2.61±0.50^&^ |
| CD4^-^CD8^-^T_CM_ (P6) |  | 2.41±0.24 | 2.38±0.09 | 2.57±0.25 |
| CD4^+^T_0_ (P1) |  | 4.32±0.69^**^ | 3.87±0.16 | 6.14±2.38 |
| CD4^+^T_N_ (P2) |  | 36.03±7.07^*^ | 31.96±7.54 | 22.00±4.91^#,&^ |
| CD4^+^T_EM_ (P0) |  | 21.89±1.32^***^ | 29.90±2.95^^^ | 20.42±1.51^&^ |
| CD8^+^T_N_ (P8) |  | 4.36±2.03^*^ | 6.35±3.54 | 0.24±0.02^&^ |
| CD8^+^T_N_L (P3) |  | 9.81±2.54 | 8.23±2.81 | 16.00±3.68 |
| CD8^+^T_EM_ (P5) |  | 15.98±5.07^**^ | 12.18±4.48 | 25.40±7.78^#,&&^ |
| CD8^+^T_CM_ (P7) |  | 3.02±0.48 | 2.68±0.68 | 4.25±0.53 |
| **C. Day 7 after re-challenge** | | | | |
| CD4^-^CD8^-^T_N_ (P9) |  | 2.27±0.4^***^ | 0.67±0.09^^^^^ | 0.55±0.07^###^ |
| CD4^-^CD8^-^T_EM_ (P4) |  | 0.91±0.14 | 1.38±0.16 | 1.32±0.06 |
| CD4^-^CD8^-^T_CM_ (P6) |  | 2.84±0.78^**^ | 1.29±0.10^^^^^ | 1.29±0.13^###^ |
| CD4^+^T_0_ (P1) |  | 13.03±2.20^***^ | 2.02±0.16^^^^ | 1.20±0.12^###,&^ |
| CD4^+^T_N_ (P2) |  | 31.33±2.03^***^ | 32.02±0.72 | 26.10±1.26^###,&&&^ |
| CD4^+^T_EM_ (P0) |  | 28.84±0.96^***^ | 35.48±2.72^^^^ | 35.33±0.36^###^ |
| CD8^+^T_N_ (P8) |  | 14.31±1.29 | 15.07±0.85 | 17.20±0.69 |
| CD8^+^T_N_L (P3) |  | 0.83±0.15^*^ | 0.68±0.09 | 0.52±0.10 |
| CD8^+^T_EM_ (P5) |  | 4.91±1.11^***^ | 7.29±1.17^^^^^ | 12.37±1.39^###,&&&^ |
| CD8^+^T_CM_ (P7) |  | 0.61±0.19^***^ | 4.11±0.56^^^^^ | 4.13±0.19^###^ |

C57BL/6 mice were immunized with p2/4 or nano2/4 on day 0 and day 21, infected with *T. cruzi* on day 42 and euthanized on day 52 and day 63 corresponding to day 10 and day 21 post-infection, respectively. Some mice in each group were re-challenged on day 63 and euthanized on day 70 (corresponding to 7 days after re-challenge). Non-infected and non-vaccinated/infected mice were used as controls. Splenocytes were labeled with fluorescent-conjugated antibodies and analyzed by flow cytometry. FlowSOM software was used to generate consensus clusters with splenic 1x10^5^ down-sampled CD3^+^ splenocytes (*n* ≥ 5 mice per group) that identified 10 T-cell sub-populations. Mean percentages (±SEM) of 10 sub-populations of T cells in all four groups of mice were obtained from at least 5 mice per group. Significance was calculated by student’s t-test (^*^ no infection vs. *Tc*) and 1-way ANOVA/Tukey’s (^^^*Tc* vs. p2/4.*Tc*, ^#^*Tc* vs. nano2/4*.Tc* and ^&^ p2/4.*Tc* vs. nano2/4.*Tc*). The *p* values of <0.05, <0.01, and <0.001 are annotated with one, two, and three symbols, respectively. Abbreviations: T_N_, Naïve T cells; T_CM_, central memory T cells; T_EM_, effector/effector memory T cells. ^a^ Control group data represents the average values of non-treated/non-infected mice collected at the three time-points (n ≥ 3-5 mice per time-point).

**S3 Table.** Median fluorescence intensity of intracellular cytokines in infected mice (± nanovaccine) at 10 days **(A)** and 21 days **(B)** post-infection, and at 7 days after re-challenge infection **(C)**.

| **Populations** | **Marker** | | **Control ^a^** | | ***T. cruzi*** | | **p2/4.*Tc*** | **nano2/4.*Tc*** |
| --- | --- | --- | --- | --- | --- | --- | --- | --- |
| **A. Day 10 post-infection** | | | | | |  |  |  |
| CD4^-^CD8^-^T_EM_ (P4) | | IFN-γ | 30.16±5.51 | | 39.28±3.61 | | 34.92±5.46 | 41.59±1.10 |
| CD4^-^CD8^-^T_CM_ (P6) | | IFN-γ | -43.88±66.18 | | -362.02±33.28 | | -242.70±21.73 | -212.20±19.28 |
| CD4^+^T_EM_ (P0) | | IFN-γ | 23.78±4.48 | | 48.82±25.19 | | 128.82±31.33^^^ | 160.07±13.51^##^ |
| CD8^+^T_EM_ (P5) | | IFN-γ | 17.13±1.46 | | 26.69±2.54^**^ | | 23.75±2.54 | 27.84±0.99 |
| CD8^+^T_CM_ (P7) | | IFN-γ | -24.85±64.15 | | -193.82±34.42 | | -207.89±34.0 | -214.40±15.96 |
| CD4^-^CD8^-^T_EM_ (P4) | | PFN | 52.70±6.11 | | 116.14±9.03^***^ | | 84.26±6.14^^^^ | 123.20±3.81^&&&^ |
| CD4^-^CD8^-^T_CM_ (P6) | | PFN | 60.55±10.80 | | 89.08±5.86^*^ | | 82.18±6.71 | 124.60±4.46^###/&&&^ |
| CD4^+^T_EM_ (P0) | | PFN | 57.84±9.35 | | 106.29±7.81^**^ | | 87.60±6.67 | 123.30±2.97^&&^ |
| CD8^+^T_EM_ (P5) | | PFN | 28.85±5.16 | | 104.81±8.78^***^ | | 80.83±6.14^^^ | 125.00±3.04^&&&^ |
| CD8^+^T_CM_ (P7) | | PFN | 64.73±8.23 | | 104.63±5.61^***^ | | 95.14±7.28 | 148.20±3.42^###/&&&^ |
| CD4^-^CD8^-^T_EM_ (P4) | | GZB | 81.28±7.01 | | 146.35±13.05^***^ | | 114.96±7.13 | 131.30±5.22 |
| CD4^-^CD8^-^T_CM_ (P6) | | GZB | 86.51±7.32 | | 91.23±4.26 | | 107.50±8.18 | 122.90±5.48^##^ |
| CD4^+^T_EM_ (P0) | | GZB | 61.78±4.54 | | 89.63±4.36^***^ | | 90.69±5.88 | 102.70±3.00 |
| CD8^+^T_EM_ (P5) | | GZB | 57.64±6.75 | | 159.39±14.97^**^ | | 111.78±8.07 | 132.80±5.24 |
| CD8^+^T_CM_ (P7) | | GZB | 68.94±3.92 | | 103.70±6.76^***^ | | 117.31±5.71 | 139.00±5.30^###/&^ |
| **B. Day 21 post-infection** | | | |  |  |  |  |  |
| CD4^-^CD8^-^T_EM_ (P4) | | IFN-γ |  | | 80.21±10.04^***^ | | 78.18±16.70 | 98.74±10.65 |
| CD4^-^CD8^-^T_CM_ (P6) | | IFN-γ |  | | -14.63±73.73 | | 115.40±16.74^^^^^ | 154.00±28.12^###/&&&^ |
| CD4^+^T_EM_ (P0) | | IFN-γ |  | | 91.47±24.39 | | 91.03±38.32 | 150.00±17.54 |
| CD8^+^T_EM_ (P5) | | IFN-γ |  | | 65.98±6.23^***^ | | 93.60±6.88 | 140.96±19.25^###/&&^ |
| CD8^+^T_CM_ (P7) | | IFN-γ |  | | -13.25±81.39 | | 220.80±38.96^^^^^ | 202.20±26.87^###^ |
| CD4^-^CD8^-^T_EM_ (P4) | | PFN |  | | 146.75±9.37^***^ | | 116.42±16.25 | 167.80±14.26^&&^ |
| CD4^-^CD8^-^T_CM_ (P6) | | PFN |  | | 129.84±7.11^***^ | | 103.54±17.74 | 166.20±10.21^#/&&&^ |
| CD4^+^T_EM_ (P0) | | PFN |  | | 87.88±6.13^*^ | | 164.70±7.38^^^^^ | 179.40±8.94^###^ |
| CD8^+^T_EM_ (P5) | | PFN |  | | 129.08±9.74^***^ | | 143.22±12.45 | 185.60±19.07^##/&^ |
| CD8^+^T_CM_ (P7) | | PFN |  | | 85.13±8.95 | | 130.74±17.89 | 204.40±8.79^###/&&^ |
| CD4^-^CD8^-^T_EM_ (P4) | | GZB |  | | 90.14±11.75 | | 70.38±0.74 | 76.56±7.19 |
| CD4^-^CD8^-^T_CM_ (P6) | | GZB |  | | 68.99±6.19 | | 56.50±1.95 | 71.16±4.04^&^ |
| CD4^+^T_EM_ (P0) | | GZB |  | | 66.91±5.60 | | 67.84±2.98 | 69.84±3.34 |
| CD8^+^T_EM_ (P5) | | GZB |  | | 85.14±17.27 | | 151.10±13.11^^^ | 155.84±14.58^#^ |
| CD8^+^T_CM_ (P7) | | GZB |  | | 64.16±5.87 | | 68.30±1.88 | 84.80±3.53^###/&&^ |
| **C. Day 7 after re-challenge** | | | | |  |  |  |  |
| CD4^-^CD8^-^T_EM_ (P4) | | IFN-γ |  | | 38.95±9.14 | | 44.08±2.38 | 47.28±2.19 |
| CD4^-^CD8^-^T_CM_ (P6) | | IFN-γ |  | | -88.7±10.29 | | -166.33±3.86 | -168.67±13.57 |
| CD4^+^T_EM_ (P0) | | IFN-γ |  | | 56.45±3.76^**^ | | 103.87±5.19^^^ | 124.62±4.66^###/&^ |
| CD8^+^T_EM_ (P5) | | IFN-γ |  | | 17.13±1.46 | | 117.88±5.96^^^^^ | 127.12±5.89^###^ |
| CD8^+^T_CM_ (P7) | | IFN-γ |  | | -9.83±19.92 | | -116.07±8.34 | -108.17±13.27 |
| CD4^-^CD8^-^T_EM_ (P4) | | PFN |  | | 65.05±3.98 | | 128.17±2.63^^^^^ | 176.00±3.20^###/&&&^ |
| CD4^-^CD8^-^T_CM_ (P6) | | PFN |  | | 60.57±4.37 | | 115.50±1.63^^^^^ | 161.00±3.28^###/&&&^ |
| CD4^+^T_EM_ (P0) | | PFN |  | | 59.3±1.46 | | 129.50±1.89^^^^^ | 172.50±2.97^###/&&&^ |
| CD8^+^T_EM_ (P5) | | PFN |  | | 59.95±3.55^*^ | | 109.33±1.67^^^ | 151.50±3.49^###/&^ |
| CD8^+^T_CM_ (P6) | | PFN |  | | 63.35±2.82 | | 144.33±1.94^^^^^ | 194.17±3.58^###/&&&^ |
| CD4^-^CD8^-^T_EM_ (P4) | | GZB |  | | 72.12±19.29 | | 94.92±2.79 | 116.83±5.47^##/&&^ |
| CD4^-^CD8^-^T_CM_ (P6) | | GZB |  | | 37.87±3.50^***^ | | 76.22±3.10^^^^^ | 98.35±4.29^###/&&&^ |
| CD4^+^T_EM_ (P0) | | GZB |  | | 35.3±2.58^**^ | | 78.57±2.92^^^ | 102.33±4.12^###/&^ |
| CD8^+^T_EM_ (P5) | | GZB |  | | 40.17±7.64 | | 77.93±2.87^^^^^ | 96.67±5.45^###/&&^ |
| CD8^+^T_CM_ (P7) | | GZB |  | | 51.8±5.82^*^ | | 86.52±3.61^^^ | 110.33±4.08^###/&^ |
| C57BL/6 female mice were immunized, infected and euthanized as described in S2 Table. Splenocytes were analyzed by flow cytometry. The median fluorescent intensities of IFN-γ and markers of T cell cytotoxicity (perforin (PFN) and granzyme B (GZB) in T effector/effector memory (T_EM_) and T central memory (T_CM_) subsets are presented as mean value ± SEM and derived from *n* ≥ 5 mice per group (at least duplicate observations per sample). Significance was calculated as described in S2 Table, and plotted as * (control vs. infected), (^*Tc* vs. p2/4.*Tc*, ^#^*Tc* vs. nano2/4.*Tc* and & p2/4.*Tc* vs. nano2/4.*Tc*). The p values of <0.05, <0.01, and <0.001 are annotated with one, two, and three symbols, respectively. ^a^ Control group data represents the average values of non-treated/non-infected mice collected at the three time-points (n ≥ 3-5 mice per time-point). | | | | | | | | |
